# Cover Crop Species Composition Alters the Soil Bacterial Community in a Continuous Pepper Cropping System

**DOI:** 10.3389/fmicb.2021.789034

**Published:** 2022-01-03

**Authors:** Huan Gao, Gangming Tian, Muhammad Khashi u Rahman, Fengzhi Wu

**Affiliations:** ^1^Department of Horticulture and Landscape Architecture, Northeast Agricultural University, Harbin, China; ^2^Key Laboratory of Biology and Genetic Improvement of Horticultural Crops (Northeast Region), Ministry of Agriculture and Rural Affairs, Northeast Agricultural University, Harbin, China

**Keywords:** cover crop, bacterial community, continuous cropping, pepper, bacterial diversity

## Abstract

Cover crops can improve soil biological health and alter the composition of soil microbial communities in agricultural systems. However, the effects of diversified cover crops on soil microbial communities in continuous cropping systems are unclear. Here, using different soil biochemical analysis, quantitative PCR and 16S rRNA amplicon sequencing, we investigated the effects of cover crops, alone or in mixture, on soil physicochemical properties in 2019 and 2020, and soil bacterial communities in 2020 in a continuous pepper cropping system. A field trial was established before pepper planting and eight treatments were included: fallow (no cover crop; CK); three sole cover crop treatments: wheat (*Triticum aestivum* L.; W), faba bean (*Vicia faba* L.; B), and wild rocket (*Diplotaxis tenuifolia*; R); and four mixed treatments: wheat + wild rocket (WR), wheat + faba bean (WB), wild rocket + faba bean (RB), and wheat + wild rocket + faba bean (WRB). The pepper yield was increased in the WR and WB in 2019 and 2020, and in the WRB in 2020. Cover crops increased the soil pH, but decreased the concentrations of NH_4_^+^ and available phosphorus. Bacterial abundance was increased by cover crop treatments, and community structure was altered in the W, WB, and WRB treatments. Moreover, we found that pH was the key factor associated with the changes in the abundance and structure of the bacterial community. Cover crop treatments altered the bacterial community structure with shifts in the dominant genera, which have plant-growth-promoting and/or pathogen-antagonistic potentials, e.g., increased the abundances of *Streptomyces*, *Arthrobacter*, and *Bacillus* spp. in the W and WRB, and *Gaiella* spp. in the WB. Overall, we found that cover crops altered the soil physicochemical properties and bacterial community, and these changes varied with species composition of the cover crops, with wheat and its combination with legumes as most effective treatments. These results suggest that the diversification within cover crops could provide better crop yield stimulatory affects by altering soil biochemical environment.

## Introduction

Modern agricultural crop production is usually focused on crop monocultures or minimum rotations, which usually negatively affect soil quality (e.g., imbalances in soil nutrition, decrease in soil microbial diversity, and the accumulation of autotoxic compounds) as well as crop yield (Gao et al., 2017). This is a negative plant-soil feedback phenomenon that has been described as “soil sickness.” Therefore, integrated soil management practices, e.g., increase in plant diversity in the field such as crop rotation, intercropping and cultivation of cover crop, have been used to alleviate soil sickness (Huang et al., 2013).

Cover crops are defined as the crops, which are grown primarily to cover and protect the soils from erosion and nutrient losses in between the period of main crop cultivation (Sarrantonio and Gallandt, 2003). Studies have shown that cover crops can be used as living mulch, green manure, and biofumigant (Snapp et al., 2005; Schipanski et al., 2014). Legumes, brassicas, cereals, and other broadleaf crops can be used as cover crops (Snapp et al., 2005; Schipanski et al., 2014). The cultivation of legumes as a cover crop is effective to improve soil N availability and moisture retention (Sullivan et al., 1991). Brassicaceae species, as cover crops, have been shown to control various soilborne diseases, and improve soil characteristics and crop yield (Larkin et al., 2010). Cereals as cover crops provide many benefits, for example, these crops increase soil fertility and organic carbon (Rorick and Kladivko, 2016), improve soil water retention (Villamil et al., 2006), and decrease bulk density (Moore et al., 2014). Overall, the use of cover crops during the fallow period in the field has the potential to improve the soil environment and increase crop productivity (Guo et al., 2008; Tian et al., 2011).

Soil microorganisms are critically important for maintaining soil functions and ecosystem sustainability because they are involved in the cycling of nutrients and the turnover of organic matter (Liu et al., 2020a). Studies have shown that cover crops can alter the dynamics of soil bacterial and fungal communities (Patkowska et al., 2016), stimulate beneficial microorganisms (Berendsen et al., 2012), and suppress soilborne pathogens (Manici et al., 2004). Cover crops vary in their characteristics and can have diverse effects on soil microbial community composition and functions (Thapa et al., 2021). For example, oats with dense rooting systems as cover crops can increase the abundance of arbuscular mycorrhizal fungi compared with the fallow field (Kabir and Koide, 2002). Legumes with high nitrogen (N) content can promote the proliferation of microbes as compared to non-leguminous cover crops (Murungu et al., 2011). Soil substrate availability and quality are related to plant diversity and can drive changes in soil microbial community structure (Thapa et al., 2021). Mixtures of cover crop species can vary in their effects on substrates, alter environmental conditions, and increase the diversity of microbial communities (Spedding et al., 2004). For example, grass cover crops mixed with legumes promote microbial activity because of their complementary effects on residue decomposition (Njeru et al., 2014). Diverse mixtures of cover crops increase the size of total microbial community and fungal community as compared to fallow (Thapa et al., 2021). However, the effect of multispecies cover crops on microbial community in continuous cropping systems remain poorly understood.

In this study, we evaluated the effects of three different cover crops (single as well as in mixtures) on main crop yield, soil physicochemical properties, and bacterial communities in the soils of continuous pepper cropping systems. Soil bacterial community composition and abundance were analyzed by 16S rRNA amplicon sequencing and quantitative PCR, respectively. The important carbon resources for soil microorganisms from plant root exudates and litter decomposition, and the chemical composition of these substances differ among plant species (Fierer et al., 2007; Eilers et al., 2010; Birouste et al., 2012). Therefore, we hypothesized that increased cover crop diversity would lead to higher soil bacterial community diversity and abundances. Moreover, since the soil bacterial communities are sensitive to soil environmental factors (Kuramae et al., 2011; Shen et al., 2013), we also hypothesized that cover crops may affect soil bacterial communities by altering the physicochemical properties of the soil.

## Materials and Methods

### Study Site and Treatments

The experiment was conducted from March 2019 to October 2020 at the Horticultural Experimental Station of Northeast Agricultural University (45°41′N, 126°37′E) Harbin, Heilongjiang Province, China. The soil was black soil (Mollisoil) and has been under continuous pepper cropping system for 7 years. The soil had the following features: organic matter 32.76 g kg^−1^, available phosphorus (AP) 105.90 mg kg^−1^, available potassium (AK) 222.21 mg kg^−1^, NO_3_^−^-N 131.88 mg kg^−1^, NH_4_^+^-N 16.72 mg kg^−1^, pH (1:2.5, w/v) 7.29, and EC (1:2.5, w/v) 0.64 mS cm^−2^.

The experiment was conducted using a pepper (*Capsicum annuum* L.)/cover crop rotation with a randomized block design consisting of eight treatments. There were three replicate plots for each treatment, and the size of each plot was 4.5 m^2^ (5 m × 0.9 m). Cover crops treatments consist on three sole cover crops treatments: wheat (*Triticum aestivum* L; W), faba bean (*Vicia faba* L.; B), and wild rocket (*Diplotaxis tenuifolia*; R); and four mixed cover crops treatments: wheat + wild rocket (WR), wheat + faba bean (WB), wild rocket + faba bean (RB), and wheat + wild rocket + faba bean (WRB), and a fallow treatment (no cover crops; CK).

### Pepper and Cover Crop Management

**Figure 1:**
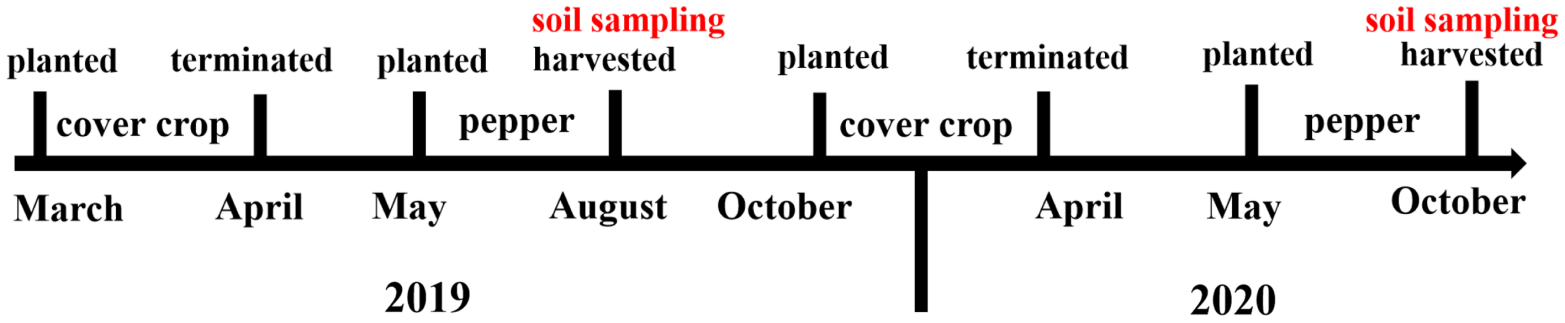
Diagram of cover crop-pepper rotation and timeline of soil sampling for soil microbial community and physicochemical properties analysis.

Cover crops were planted in fallow periods. In cover crop plots, the first phase of rotation (cover crop-pepper) began with the planting of cover crops in March 2019 and ended in April 2019. Pepper was planted in May 2019 and harvested in August 2019. The second phase of rotation (crop cover-pepper) started in October 2019 and ended in April 2020, followed by pepper planting in May 2020 and harvested in October 2020 (Figure 1).

For planting sole cover crops, 34 g of wheat, 420 g of faba bean, and 3.96 g of wild rocket seeds were sown per plot. In two or three cover crop mixture treatments, the seeding rate decreased to 50 or 33% of the monoculture rate for each species, respectively. One month later, samples of aboveground biomass were hand clipped from a 1-m^2^ area (0.20-m^2^ quadrat area at five random spots) in each plot, and dry biomass was measured after being subjected to 65°C for 72 h (Supplementary Table S1). The rest of the harvested plant residues (above-ground) were naturally dried, cut into 10-cm pieces, and incorporated into the decomposed soil. Thirty days later, the pepper seedlings at the six-leaf stage were transplanted to the greenhouse. There were two rows in each plot, and 14 pepper plants were there in one row.

### Soil Sampling

One hundred and twenty days after pepper transplantation in 2019 and 2020, bulk and rhizosphere soil samples were collected for soil physicochemical properties analysis and DNA extraction, respectively.

### Bulk Soil Sampling and Soil Physicochemical Properties Analysis

Five soil cores (8 cm diameter, 20 cm deep) were randomly collected from the inner two rows of pepper in each plot and mixed to make a composite sample – one replicate. There were three replicates of bulk soil samples for each treatment. The soils were sieved through a 2-mm mesh to remove the stones, roots, and plant debris. The soil samples were stored at −4°C for soil physicochemical analysis.

Soil EC and pH were measured in a soil-water suspension at a water/soil ratio of 2.5:1 with a conductivity meter and glass electrode, respectively. Soil ammonia nitrogen (NH_4_^+^-N) and nitrate (NO_3_^−^-N), available P (AP) and K (AK) were extracted with 2 M KCl, 1 M NaHCO_3_, and 0.5 M CH_3_COONH_4_, respectively. Then, measurements were taken from the soil extract using a Continuous Flow Analyzer (SAN++, Skalar, Breda, Netherlands; Gao et al., 2017). The concentrations of soil total nitrogen (TN) and soil organic carbon (SOC) were determined by the micro-Kjeldahl method and wet oxidation redox titration method, respectively (Lin et al., 2018).

### Rhizosphere Soil Sampling

The pepper roots were removed from the soil carefully. The soil loosely attached to pepper roots was removed by manual shaking. The soil tightly adhering to the roots was removed from the root surface by a sterile brush, sieved through a 2 mm mesh, and considered as rhizosphere soil. The rhizosphere soil samples from five plants in each treatment replicate were mixed to make a composite sample. The samples were stored at −80°C for DNA extraction; one part from 2019 and 2020 sampling each was used to determine the abundances of soil total bacteria, total fungi, *Bacillus*, and *Pseudomonas* spp., while other part was used for Illumina Miseq sequencing analysis for soil bacterial community composition.

### DNA Extraction and Quantitative PCR

Soil total DNA was extracted from rhizosphere soil using a PowerSoil DNA Isolation Kit (MO BIO Laboratories, CA, United States) according to the manufacturer’s protocols. Agarose gel electrophoretic analysis and a NanoDrop 2000 Spectrophotometer (Thermo Scientific, United States) were used to assess the quantity and quality of DNA. The abundances of soil total bacteria, fungi, *Bacillus*, and *Pseudomonas* spp. in pepper rhizosphere were estimated by quantitative PCR assays conducted with an IQ5 real-time PCR system (Bio-Rad Lab, LA, United States). For total bacteria and fungi, the primer sets 338F/518R (Jin et al., 2019) and ITS1F/ITS4 (Bellemain et al., 2010) were used to amplify the V3-V4 hypervariable region of the bacterial 16S rRNA gene and ITS1 region of the fungal ITS gene, respectively. The primer sets PsF/PsR (Garbeva et al., 2004) and BacF/BacR (Garbeva et al., 2003) were used for *Pseudomonas* and *Bacillus* spp., respectively. Melting curves and agarose gel electrophoresis were used to verify the specificity of the amplicons. Standard curves were created using 10-fold dilution series of plasmid DNA with inserted target genes. The fluorescence signal was used to calculate Ct (cycle threshold) values, using the thermocycler software. The copy number of genes in the template DNA was calculated according to the linear equation and the Ct value conversion (Henry et al., 2006).

### Illumina Miseq Sequencing and Data Processing

The compositions of soil bacterial communities in pepper rhizosphere in 2020 were analyzed with Illumina MiSeq. The V3-V4 hypervariable region of the bacterial 16S rRNA gene was amplified using the primers F338 (5′-ACTCCTACGGGAGGCAGCA-3′) and R806 (5′-ATGCAGGGACTACHVGGGTWTCTAAT-3′; Derakhshani et al., 2016). A 6-bp unique barcode was added to both the forward and reverse primers for each sample. Triplicate PCR amplifications were pooled and PCR products were purified with an Agarose Gel DNA Purification Kit (TaKaRa, China). The purified amplicons were then sequenced (2 × 300) on an Illumina Miseq platform at Majorbio Bio-Pharm Technology Co., Ltd., Shanghai, China. A total of 1,239,149 sequences were obtained from the 24 samples. The raw sequence reads were processed using the QIIME pipeline (Caporaso et al., 2010). Briefly, barcodes, adaptor sequences, and 30 low-quality bases at the end of each read were removed. Paired reads were joined (minimum overlapping read length of 20 base pairs) and quality filtered (Phred score of 20), and reads lower than 200 base pairs were removed. Chimaeras were screened and removed using USEARCH with the UCHIME algorithm (Edgar, 2013). The remaining high-quality sequences (97% similarity) were assigned to the same operational taxonomic units (OTUs) with UPARSE (Edgar, 2013). Taxonomies were assigned to the representative sequence of each OTU using the SILVA database release 128 (Quast et al., 2013). OTUs classified as chloroplasts and mitochondria, and singleton OTUs were eliminated. To avoid potential bias caused by the sequencing depth, all samples were randomly subsampled based on the lowest minimum number of sequence (20,008) per sample. The data set has been uploaded to the NCBI Sequence Read Archive (Accession Number SRP326928).

### Statistical Analysis

Data of pepper yield, soil physicochemical properties, alpha diversity indices, and microbial abundances were analyzed by one-way ANOVA. The means of different treatments were compared with Tukey’s honestly significant difference (HSD) test at the 0.05 probability level. Relative weight analysis was performed using the “relweights” package in R (Kabacoff, 2015). A multivariate regression tree (MRT) was built to identify the most important soil environmental factors affecting bacterial community diversity using the “mvpart” package in R (Yang et al., 2019). Spearman correlation coefficients were calculated to test the relationships between pepper yield or the relative abundance of genera and soil physicochemical properties in SPSS software (Version 17.0). The Shannon index was calculated by QIIME (Caporaso et al., 2010). Principal coordinate analysis (PCoA) was used to analyze the composition of the bacterial community based on the Bray–Curtis distance dissimilarity. A redundancy analysis (RDA) was conducted to evaluate the relationships between the soil environmental factors and soil bacterial communities, and Mantel tests were used to determine the effects of environmental factors on the composition of the bacterial community using the “vegan” package in R. Species indicator analysis was conducted to identify the specific OTUs in different treatments using the “indicspecies” package in R (Cáceres and Legendre, 2009), and the results were displayed using iTOL tool (Letunic and Bork, 2007).

## Results

### Pepper Yield

**Figure 2:**
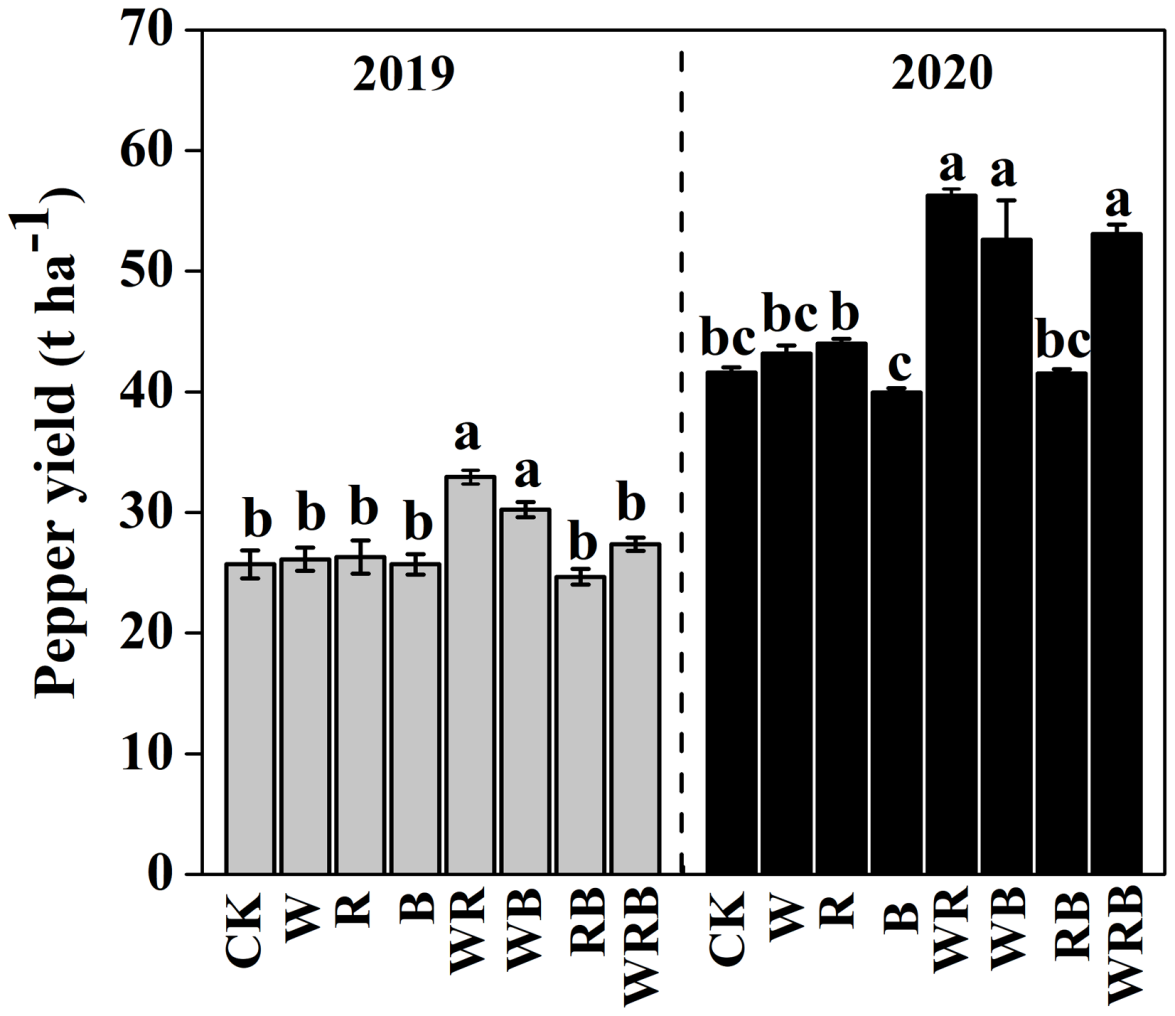
Pepper yield after cultivation of different cover crops in 2019 and 2020. CK, no cover crop; cover crop treatments: W, wheat; R, wild rocket; B, faba bean; WR, wheat + wild rocket; WB, wheat + faba bean; RB, wild rocket + faba bean; and WRB, wheat + wild rocket+faba bean. Different lowercase letters in each column denote significant differences among the samples [*p* < 0.05, one-way ANOVA; honestly significant difference (HSD) Tukey test].

Pepper yield was much higher in the WR and WB treatments than in CK in 2019, and no difference was observed among the other treatments. In 2020, pepper yield in WR, WB and WRB treatments were significantly higher than that in rest of the treatments, and significant differences were observed between the R and B treatments. The pepper yield in 2020 was higher than that in 2019 (Figure 2).

### Soil Physicochemical Properties

The soil physicochemical properties of the treatments are shown in Table 1. The lowest soil pH was observed in CK. However, the EC and the concentrations of NH_4_^+^-N and AP were decreased in the cover crop treatments compared with CK. The concentration of NO_3_^−^-N was higher in W, WR, RB, and WRB treatments than in CK in both years. The concentration of AK was higher in the W and WR treatments in 2019 and the W and B treatments in 2020 than in CK, no differences were observed among the other treatments. The content of TN was higher in the cover crop treatments than in CK in both years, with the exception of the RB treatment in both years and the B treatment in 2019. The SOM was higher in the B, WR, and WB treatments in 2019 and the WB treatment in 2020 than in CK; SOM was highest in the WB treatment and lowest in the WRB treatment. C/N was higher in CK compared with the other treatments, with the exception of the B and WB treatments in 2019. In 2020, the C/N was higher in CK than in the WRB treatment (Table 1).

**Table 1:** Soil physicochemical properties in different treatments in 2019 and 2020.

	Treatment	pH	EC (uS.cm^−1^)	NO_3_^−^ (mg kg^−1^)	NH_4_^+^ (mg kg^−1^)	TN (g kg^−1^)	AP (mg kg^−1^)	AK (mg kg^−1^)	SOM	C/N
2019	CK	6.96 ± 0.01 e	408.33 ± 20.60 a	36.68 ± 1.82 d	25.24 ± 2.52 a	3.36 ± 0.14 d	81.44 ± 7.18 a	201.67 ± 3.51 cd	61.14 ± 2.60 cd	18.17 ± 0.10 b
	W	7.06 ± 0.01 bc	301.33 ± 22.50 b	49.88 ± 2.18 abc	20.32 ± 1.42 b	3.76 ± 0.06 ab	42.10 ± 2.23 b	227.33 ± 3.21 a	61.87 ± 1.03 bcd	16.45 ± 0.04 e
	R	7.06 ± 0.01 bc	281.33 ± 18.45 b	45.25 ± 2.29 c	20.79 ± 1.42 b	3.63 ± 0.04 bc	47.63 ± 2.10 b	211.67 ± 4.62 bc	63.82 ± 0.69 abc	17.58 ± 0.02 d
	B	7.03 ± 0.01 d	256.33 ± 11.68 bc	44.14 ± 1.85 c	19.68 ± 0.72 bc	3.50 ± 0.02 cd	46.28 ± 2.21 b	201.67 ± 3.79 cd	65.03 ± 0.34 ab	18.60 ± 0.02 a
	WR	7.08 ± 0.01 ab	258.00 ± 10.54 bc	53.28 ± 3.06 a	17.95 ± 0.84 bc	3.63 ± 0.02 bc	46.33 ± 0.82 b	216.00 ± 5.20 b	65.28 ± 0.34 ab	17.98 ± 0.02 c
	WB	7.08 ± 0.01 ab	269.33 ± 14.01 b	30.62 ± 0.37 d	18.37 ± 0.86 bc	3.63 ± 0.02 bc	43.58 ± 2.02 b	202.00 ± 1.00 c	67.47 ± 0.34 a	18.59 ± 0.02 a
	RB	7.05 ± 0.01 cd	277.33 ± 14.05 b	46.32 ± 2.42 bc	16.60 ± 0.71 c	3.43 ± 0.03 d	46.92 ± 2.97 b	198.00 ± 2.65 c	62.60 ± 0.60 bcd	18.25 ± 0.02 b
	WRB	7.09 ± 0.01 a	222.33 ± 14.19 c	51.51 ± 2.37 ab	19.39 ± 0.87 bc	3.83 ± 0.05 a	45.73 ± 0.23 b	198.00 ± 2.66 c	59.92 ± 0.69 d	15.66 ± 0.02 f
										
2020	CK	6.91 ± 0.01 e	448.67 ± 17.24 a	36.84 ± 0.18 d	21.04 ± 0.82 a	3.27 ± 0.02 c	59.45 ± 1.98 a	221.67 ± 7.09 c	60.30 ± 2.17 bc	18.46 ± 0.65 ab
	W	7.08 ± 0.01 bc	247.33 ± 15.31 b	51.97 ± 4.65 a	18.94 ± 0.60 b	3.73 ± 0.04 a	49.74 ± 0.46 bc	262.33 ± 4.51 a	61.78 ± 0.60 abc	16.55 ± 0.16 bc
	R	7.08 ± 0.01 bc	230.67 ± 12.86 b	43.90 ± 2.07 bcd	18.75 ± 0.76 b	3.54 ± 0.15 ab	49.29 ± 0.86 c	236.67 ± 9.61 bc	63.50 ± 1.84 abc	17.97 ± 0.52 ab
	B	7.05 ± 0.01 d	172.67 ± 14.01 d	43.47 ± 1.48 cd	17.91 ± 0.21 bc	3.69 ± 0.07 ab	45.65 ± 2.01 cd	248.67 ± 7.02 ab	66.70 ± 1.25 ab	18.05 ± 0.34 ab
	WR	7.12 ± 0.01 a	174.67 ± 11.15 cd	50.92 ± 2.06 ab	18.05 ± 0.28 bc	3.60 ± 0.01 ab	48.64 ± 1.33 c	224.33 ± 9.71 c	64.24 ± 3.32 abc	17.84 ± 0.92 ab
	WB	7.11 ± 0.01 ab	229.67 ± 17.56 b	29.17 ± 1.28 e	17.62 ± 0.57 bc	3.60 ± 0.20 ab	43.58 ± 2.02 d	237.33 ± 7.51 bc	67.43 ± 1.94 a	18.74 ± 0.48 a
	RB	7.07 ± 0.01 cd	218.33 ± 17.79 bc	45.65 ± 1.99 abc	15.78 ± 0.57 d	3.47 ± 0.16 bc	49.14 ± 1.09 c	221.33 ± 8.08 c	62.02 ± 2.85 abc	17.89 ± 0.82 ab
	WRB	7.12 ± 0.01 a	138.00 ± 18.73 d	50.10 ± 4.03 abc	16.48 ± 0.39 cd	3.73 ± 0.04 a	53.58 ± 1.22 b	240.67 ± 7.64 abc	59.07 ± 0.35 c	15.82 ± 0.09 c

*Different letters in each column indicate significant differences among the samples (p < 0.05, HSD Tukey test). TN, soil total nitrogen; AP, available phosphorus; AK, available potassium; and SOM, soil organic matter. CK, no cover crop; W, wheat; R, wild rocket; B, faba bean; WR, wheat + wild rocket; WB, wheat + faba bean; RB, wild rocket + faba bean; and WRB, wheat + wild rocket + faba bean*.

### Abundances of Bacteria, Fungi, *Pseudomonas*, and *Bacillus* spp.

**Figure 3:**
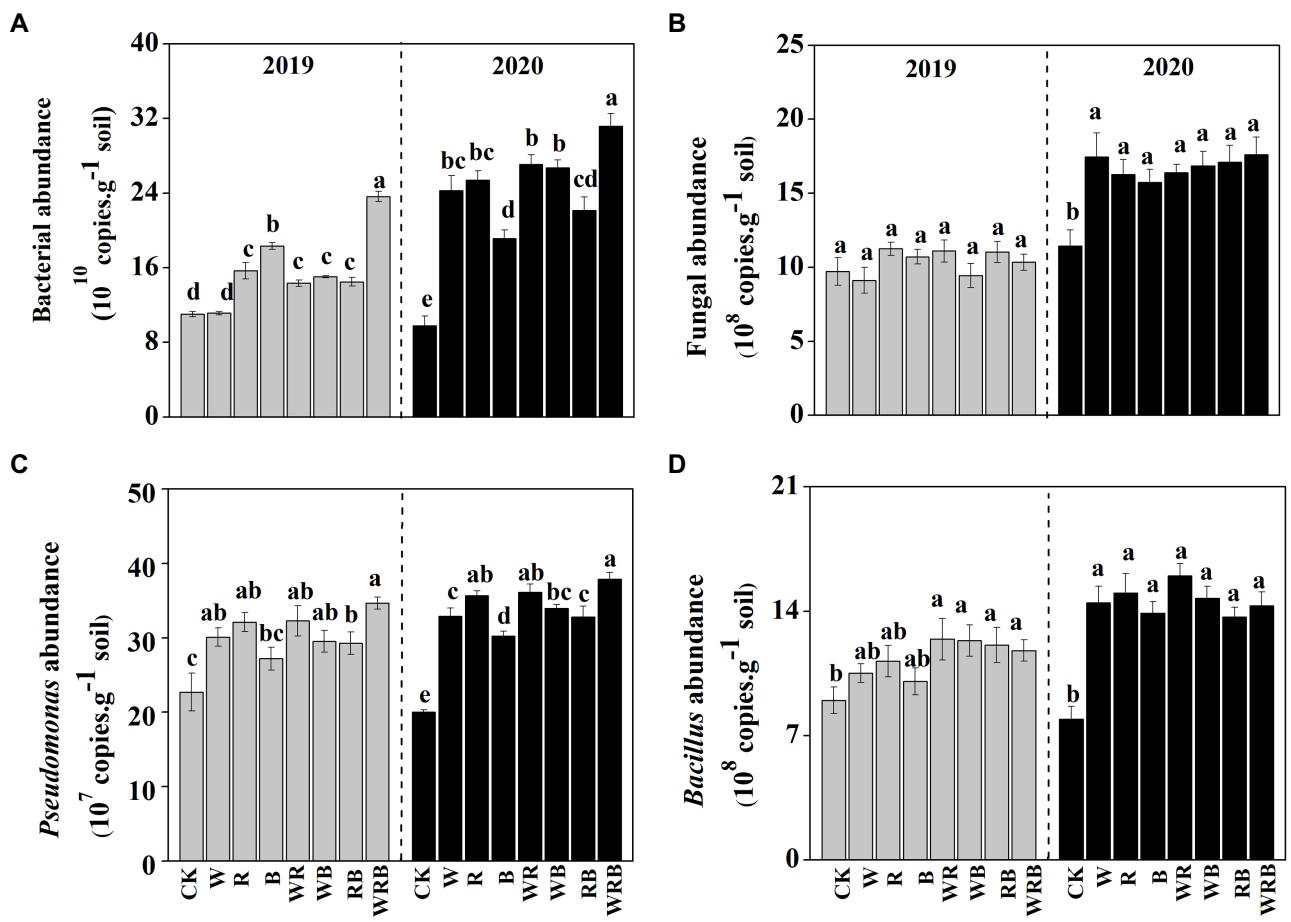
The abundances of total bacteria **(A)**, total fungi **(B)**, *Pseudomonas* spp. **(C)**, and *Bacillus* spp. **(D)** in 2019 and 2020 in the pepper rhizosphere cultivated after different cover crops. CK, no cover crop; W, wheat; R, wild rocket; B, faba bean; WR, wheat + wild rocket; WB, wheat +faba bean; RB, wild rocket + faba bean; and WRB, wheat + wild rocket + faba bean. Different lowercase letters in each column denote significant differences among the samples (*p* < 0.05; HSD Tukey test).

**Figure 4:**
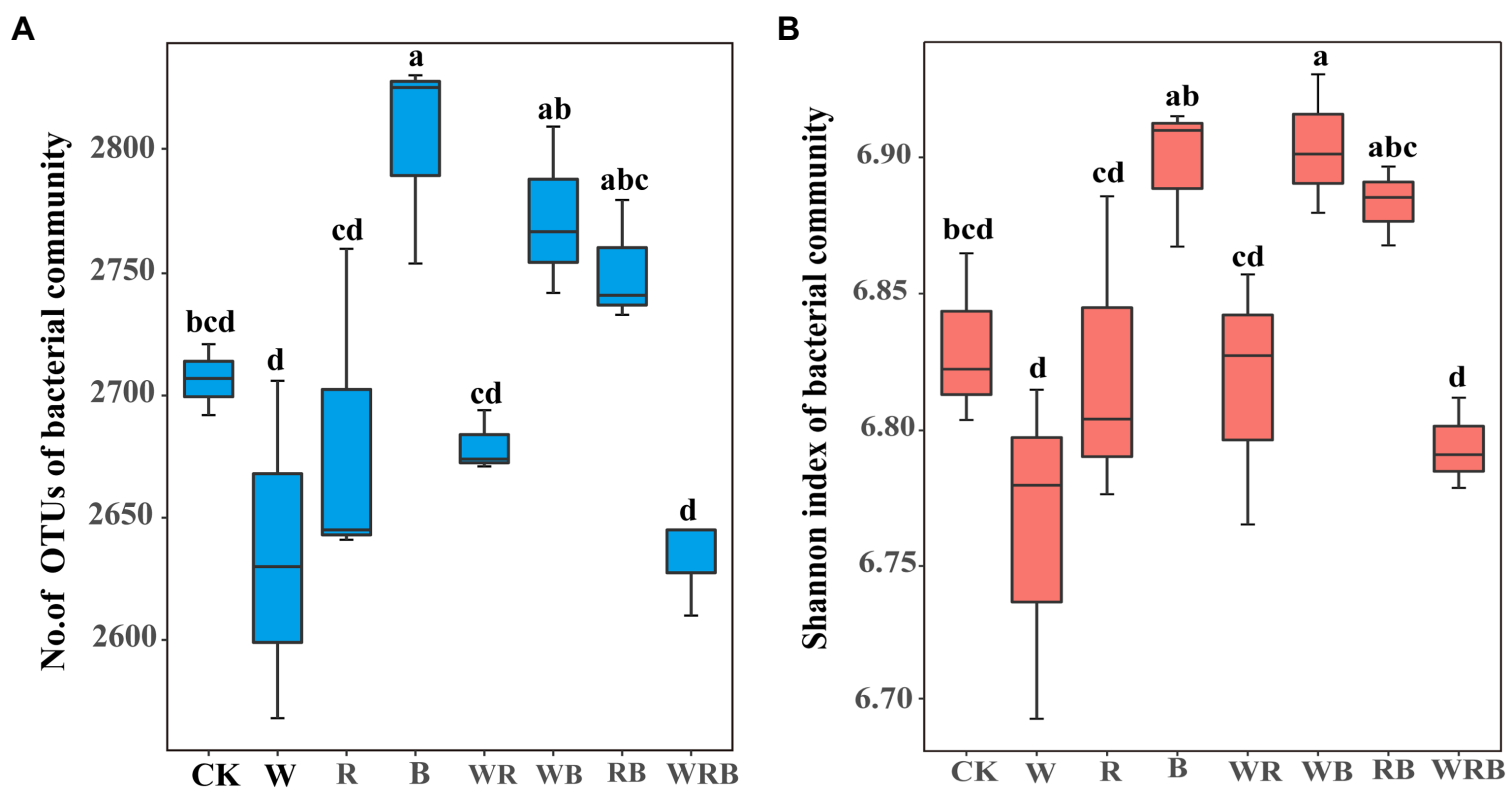
Relative influence of soil physicochemical properties on abundance of bacteria was evaluated using relative weight analysis **(A)**. Multivariate regression tree (MRT) analysis of alpha diversity (observed species, Chao 1, Shannon and Simpson indexes) of bacterial communities and soil physicochemical properties **(B)**. Treatments and the number of samples included in the analysis are shown under bar plots. AK, available potassium; AP, available phosphorus; C/N, carbon to nitrogen ratio; SOM, soil organic matter; and TN, total nitrogen.

The abundances of bacteria were significantly higher in the cover crop treatments than in CK in 2019 and 2020, with the exception of the W treatment in 2019, and the abundance of bacteria was the highest in the WRB treatment (Figure 3A). The abundance of fungi was higher in all cover crop treatments in 2020 than in CK, while no difference among treatment was found in 2019 (Figure 3B). Cover crop treatments significantly increased the abundance of *Pseudomonas* in 2019 and 2020, with the exception of the B treatment in 2019 (Figure 3C). The abundance of *Bacillus* was significantly higher in cover crop treatments than in CK, with the exception of the W, R, and B treatments in 2019 (Figure 3D). Relative weight analysis showed that soil pH was the major factor affecting the abundance of bacteria, and it explained approximately 39.43% of the variation in the 16SrRNA gene copy number (Figure 4A).

### Bacterial Community Diversity and Composition

**Figure 5:**
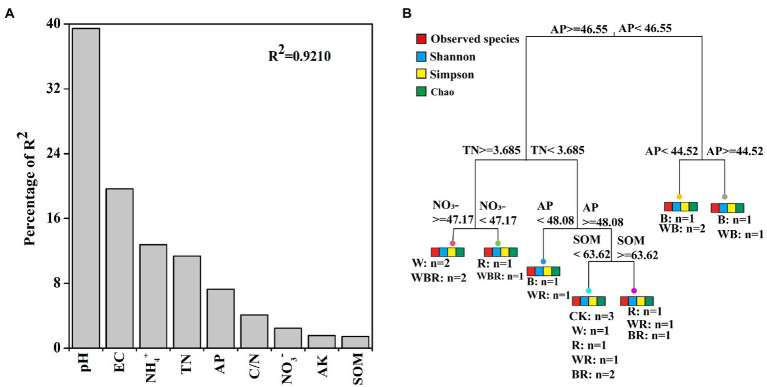
The number of operational taxonomic units (OTUs) **(A)** and Shannon index **(B)** of pepper rhizosphere bacterial communities in different treatments. CK, no cover crop; W, wheat; R, wild rocket; B, faba bean; WR, wheat + wild rocket; WB, wheat + faba bean; RB, wild rocket + faba bean; and WRB, wheat + wild rocket + faba bean. Different lowercase letters in each column denote significant differences among the samples (*p* < 0.05; HSD Tukey test).

The number of OTUs of the bacterial community was significantly increased in the B treatment compared with CK (Figure 5A). The Shannon index of the bacterial community was significantly higher in the WB treatment than in CK (Figure 5B). MRT analysis revealed that bacterial diversity was mainly shaped by soil available P (Figure 4B).

**Figure 6:**
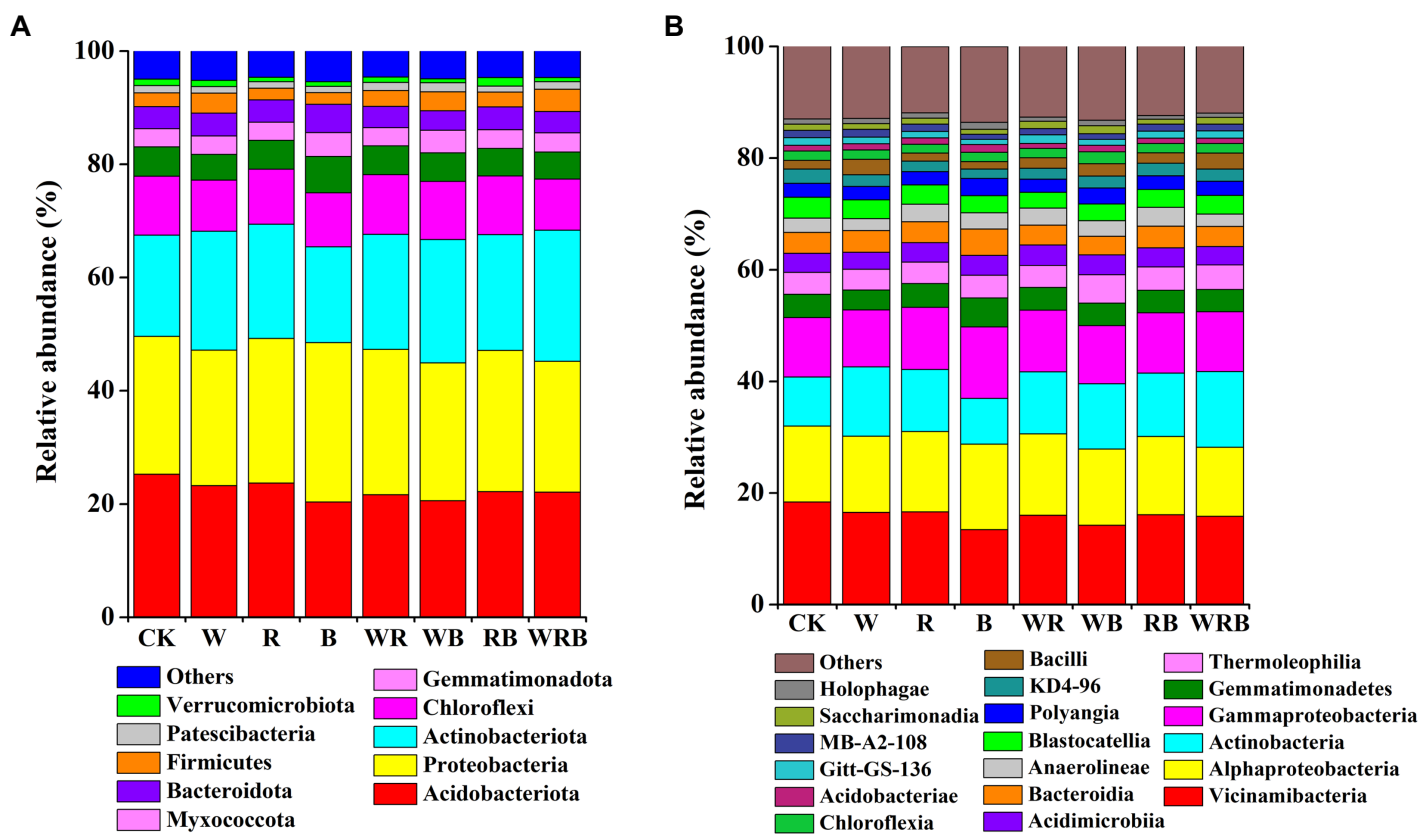
Relative abundance of dominant bacterial phyla **(A)** and classes **(B)** in pepper rhizosphere. Bacterial phyla and classes with average relative abundance >1% in at least one treatment were shown. CK, no cover crop; W, wheat; R, wild rocket; B, faba bean; WR, wheat + wild rocket; WB, wheat + faba bean; RB, wild rocket + faba bean; and WRB, wheat + wild rocket + faba bean.

Actinobacteriota, Acidobacteriota, Proteobacteria, Chloroflexi, Gemmatimonadota, Bacteroidota, and Myxococcota were the dominant phyla in all samples, accounting for more than 90% of the total sequences. The relative abundances of Firmicutes, Patescibacteria, and Verrucomicrobiota were low (relative abundance <4%; Figure 6A). The relative abundances of Vicinamibacteria, Alphaproteobacteria, Actinobacteria, Gammaproteobacteria, Gemmatimonadetes, Thermoleophilia, Acidimicrobiia, and Bacteroidia were higher in all soil samples (relative abundance >3%), and these classes made up more than 66% of the total bacterial sequences. Eleven other bacterial classes, including Anaerolineae, Blastocatellia, Polyangia, KD4-96, and Bacilli were less abundant (relative abundance <3%) in all soil samples (Figure 6B).

**Figure 7:**
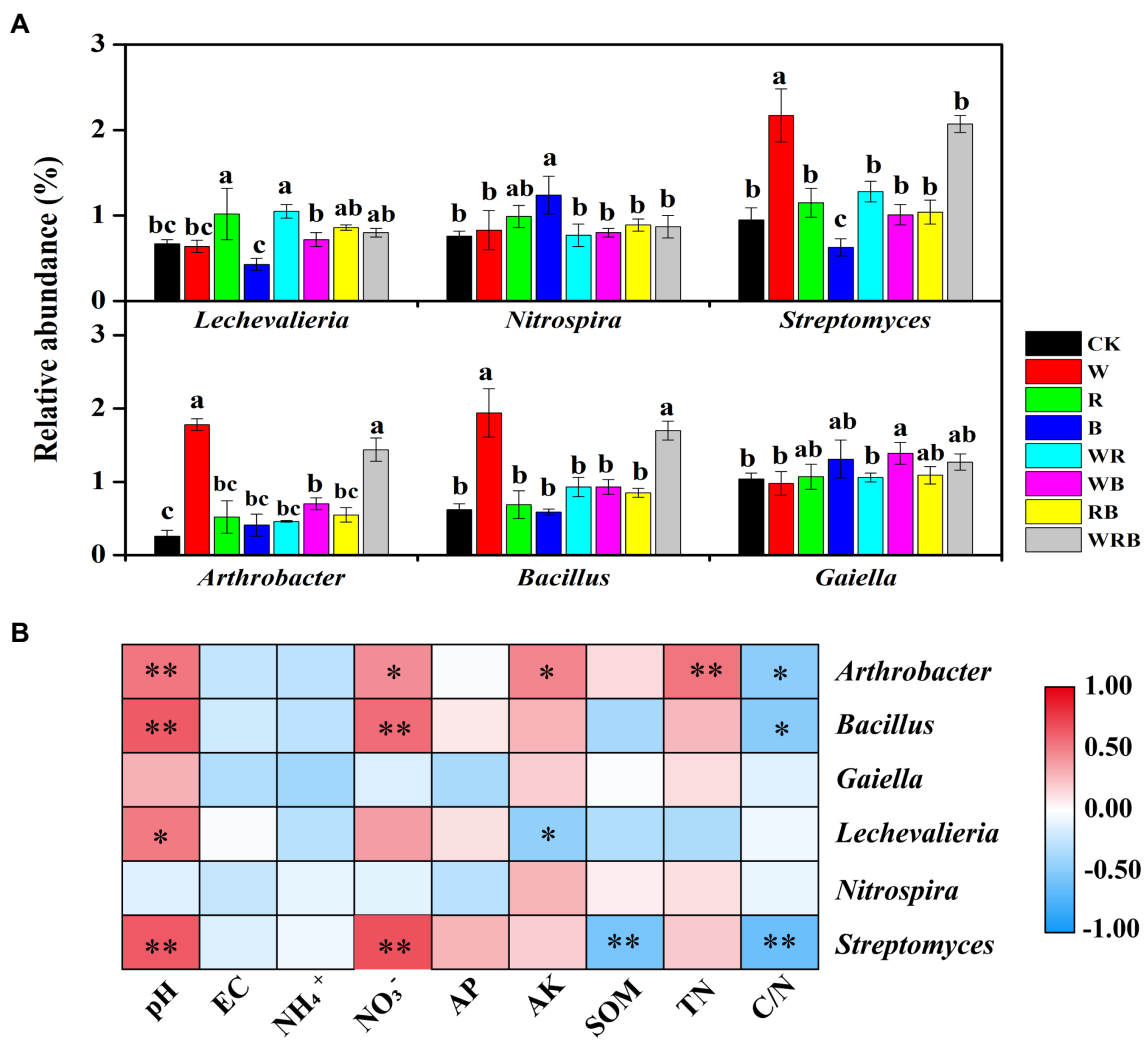
The bacterial significantly altered genera with relative abundances >1% in at least one treatment were shown. Different lowercase letters in each column denote significant differences among the samples (*p* < 0.05, HSD Tukey test; **(A)**. Spearman correlation analysis between the relative abundances of dominant bacterial genera and soil physicochemical properties. *r* indicates the correlation coefficient; ^*^p < 0.05, ^**^p < 0.01. AK, available potassium; AP, available phosphorus; C/N, carbon to nitrogen ratio; SOM, soil organic matter; and TN, total nitrogen **(B)**. CK, no cover crop; W, wheat; R, wild rocket; B, faba bean; WR, wheat + wild rocket; WB, wheat + faba bean; RB, wild rocket + faba bean; and WRB, wheat + wild rocket + faba bean.

The relative abundances of *Streptomyces*, *Arthrobacter*, and *Bacillus* were significantly increased in the W and WRB treatments compared with CK. The relative abundance of *Streptomyces* was significantly decreased in the B treatment relative to CK. The relative abundance of *Lechevalieria* was higher in the R and WR treatments than in CK. The relative abundances of *Nitrospira* and *Gaiella* were significantly increased in the WB and B treatments, respectively, compared with CK (Figure 7A). Correlation analysis indicated that soil physicochemical properties were closely correlated with the abundance of most of the genera (Figure 7B). The relative abundances of *Arthrobacter*, *Bacillus*, and *Streptomyces* were positively related to pH and NO_3_^−^, and negatively related to C/N. In contrast to *Bacillus* and *Streptomyces*, the relative abundance of *Arthrobacter* was positively related to AK and TN. The relative abundance of *Lechevalieria* was positively related to pH but negatively related to soil AK.

### Structure of the Bacterial Community and Its Correlation With Soil Properties

**Figure 8:**
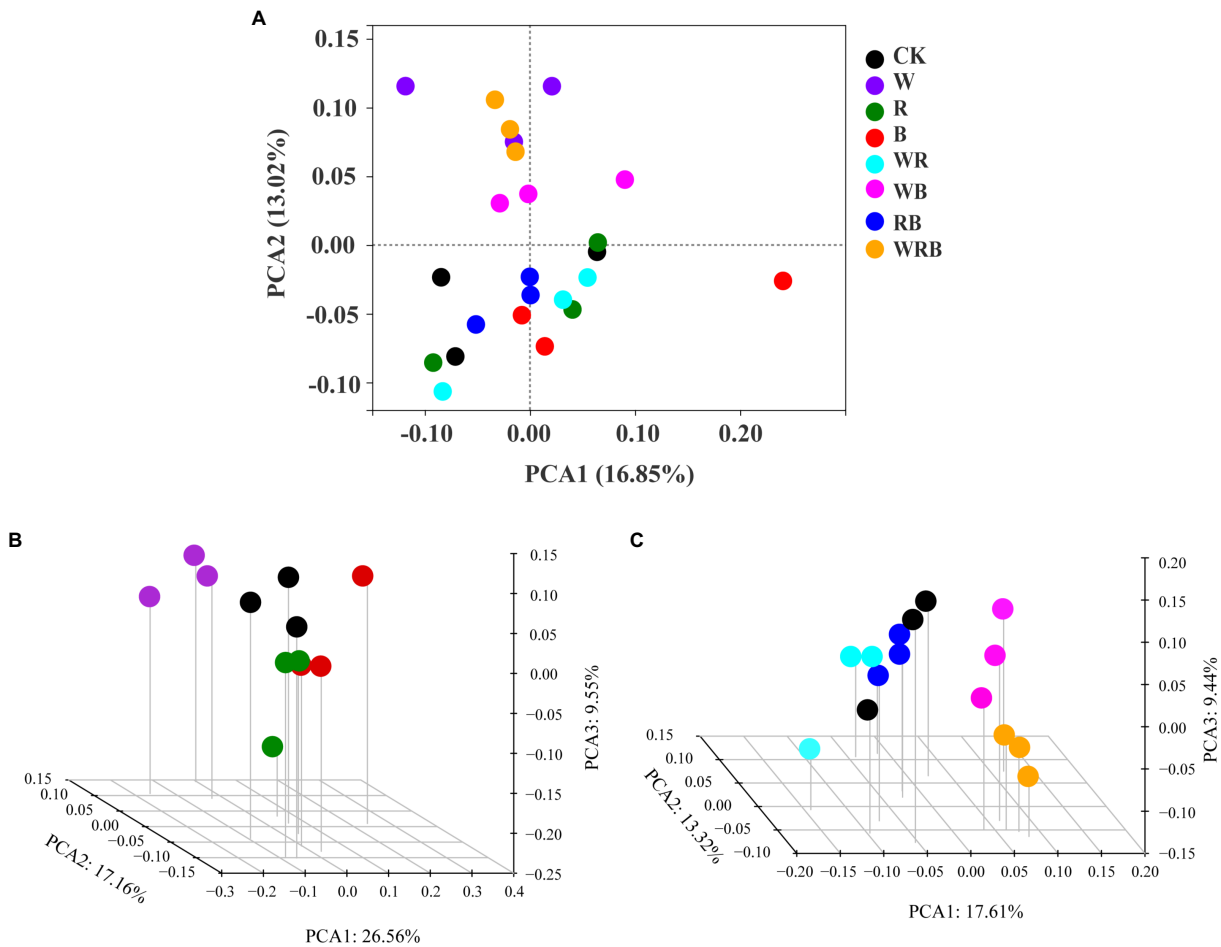
Principal Coordinates Analysis (PCoA) plot of bacterial communities based on OTUs from 24 soil samples **(A)**. The subplans of **(B)** and **(C)** indicated the separated PCoA 3D plots, which showed the response of the bacterial composition in sole cover crop treatments and in mixed species cover crop treatments, respectively. CK, no cover crop; W, wheat; R, wild rocket; B, faba bean; WR, wheat + wild rocket; WB, wheat + faba bean; RB, wild rocket + faba bean; and WRB, wheat + wild rocket + faba bean.

**Figure 9:**
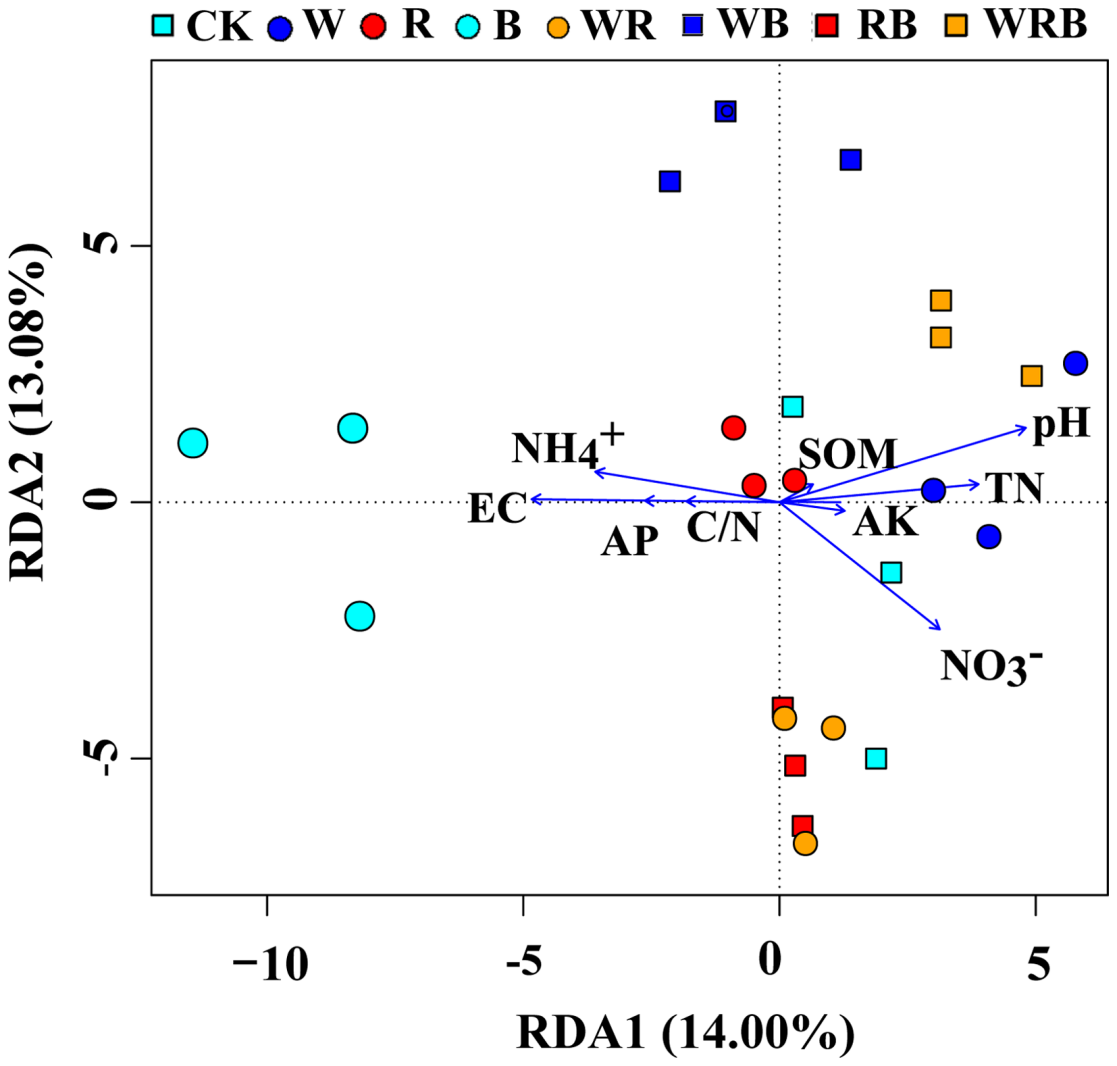
Redundancy analysis (RDA) showing correlations between soil bacterial community and soil physicochemical properties. CK, no cover crop; W, wheat; R, wild rocket; B, faba bean; WR, wheat + wild rocket; WB, wheat + faba bean; RB, wild rocket + faba bean; and WRB, wheat + wild rocket + faba bean.

Principal coordinate analysis of bacterial communities at the OTU level revealed variation in the bacterial community composition among samples; the first and second principal coordinates together explained 29.87% of the variation in community structure (Figure 8A). Among sole cover crop treatments, the community structures of the R and B treatments were most similar to that of CK, and the community structures of these treatments differed to that of the W treatment (Figure 8B). Among mixed species cover crop treatments, the community structures of the WR and RB treatments were most similar to that of CK, and the community structures of these treatments differed from the community structures of the WB and WRB treatments (Figure 8C). RDA (Figure 9) and the Mantel tests (Table 2) were conducted to determine the relationship between soil physicochemical properties and soil bacterial community composition. Among the environmental factors, pH and NH_4_^+^ were significantly correlated with the soil bacterial community composition.

**Table 2:** Mantel test correlations between bacteria community structure and soil physicochemical properties.

Soil	*r*	*p*
pH	0.343	**0.007** ^*^
EC	0.173	0.092
NO-3	0.169	0.057
NH + 4	0.254	**0.013** ^*^
AP	0.023	0.429
AK	0.005	0.459
SOM	−0.026	0.620
TN	0.128	0.094
C/N	0.027	0.365

Only significant correlations (^*^p < 0.05) were shown in bold number. TN, soil total nitrogen; AP, available phosphorus; AK, available potassium; and SOM, soil organic matter.

### Indicator Species Analysis

**Figure 10:**
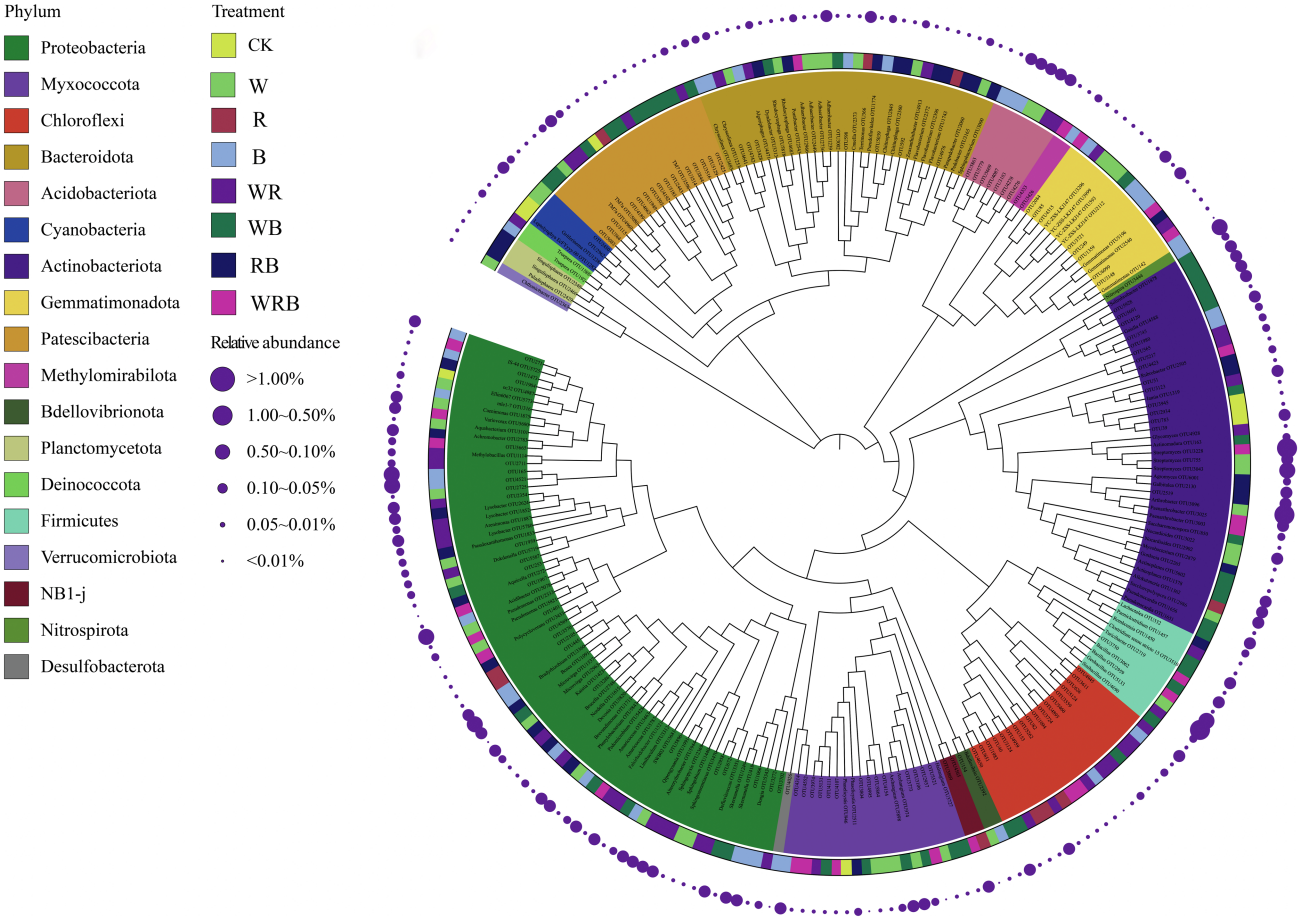
The maximum likelihood tree of the cover crops sensitive OTUs (CK, no cover crop; cover crop treatments: W, wheat; R, wild rocket; B, faba bean; WR, wheat + wild rocket; WB, wheat + faba bean; RB, wild rocket + faba bean; and WRB, wheat + wild rocket + faba bean). The background of the OTU names is colored according to their taxonomic affiliations at the phylum level. Taxa that could be assigned to the genus level were shown as genus, otherwise were shown as OTU id. The inner strip indicates the associations between sensitive OTUs and different cover crop treatments. The size of each circle represents the relative abundances of each OTU in each cover crop treatment.

Indicator species analysis was carried out to identify the important OTUs in the different cover crop treatments. We identified indicator species, which are used as ecological indicators of habitat types or communities, based on the relative abundance and frequency of species. Two hundred and sixty four OTUs were identified to be sensitive to cover crop treatment. Most of these sensitive OTUs belonged to Proteobacteria, Actinobacteriota, and Patescibacteria phyla. Only eight OTUs were enriched solely in the CK. A total of 43, 51, and 26 OTUs were enriched solely in the W, WB, and WRB treatments, respectively (Figure 10).

## Discussion

### Effect of Cover Crops on Pepper Yield and Soil Characteristics

Cover crops usually have positive effects on main crop production and soil properties (e.g., moisture and nutrient status), but their effects greatly depend on the crop species (Marinari et al., 2015). In this study, pepper yield was increased in the WR and WB treatments in 2019 and 2020 and the WRB treatment in 2020 compared with CK (Figure 2). A positive correlation between pepper yield and soil available K was found regardless of year in our study (Supplementary Table S2), which was consistent with the finding reported by Tao et al. (2016), indicating that enhanced pepper yield might result from the increase of soil nutrient elements. Furthermore, we found that the pepper production in cover crop treatments in 2020 was higher than that in 2019 (Figure 2). Previous study showed that the net effects of biodiversity on ecosystem functions grow stronger with time because the magnitude of resource complementarity increases as experiment run longer (Cardinale et al., 2012). Soil nutrient status and pH are the important indices of soil properties (Raaijmakers et al., 2009; Thiele-Bruhn et al., 2012), and the deterioration of these properties is the main cause of “soil sickness” (Chang et al., 2017). In our study, we found that cover crops decreased the soil available P content in 2 years (Table 1). This finding is consistent with the results of Gao et al. (2017) showing that lower soil available P content might stem from the higher uptake of main crops or cover crops. In contrast, Wang et al. (2021) found that long-term cover crops could improve soil P availability. These results suggest that the duration of cover crops treatment might have an important effect on soil P nutrient. In addition, we found that cover crop treatments increased soil pH as compared to CK (Table 1). Similar results were found in the study reported by Balota and Chaves (2011) where pH was increased from 4 to 6 when cultivated with different summer legumes in the coffee crop. However, Wang et al. (2021) found that legumes as cover crop decreased soil pH and it was attributed to the secretion of acids and protons from their roots (Zhou et al., 2017). In our present study, despite the general increase in soil pH after being treated by cover crops, the pH was lowest in the B treatment, and this is in agreement with previous studies showing a higher soil pH in non-legumes than in legumes (Li et al., 2007; Maltais-Landry, 2015). The concentration of NH_4_^+^ was decreased in cover crop treatments after 2 years, which might be caused by the increase in nitrification activity stemming from the higher pH in cover crop treatments, which promoted the conversion of NH_4_^+^-N to NO_3_^−^-N (Islam et al., 2006; Lin et al., 2018). Soil microorganisms are involved in soil nutrient cycling (van der Heijden et al., 2008). Therefore, cover crops might indirectly affect soil properties through plant-mediated changes in the soil microbial community.

### Effect of Cover Crops on Bacterial Abundance and Diversity

Increasing evidence indicates that cover crops can alter soil microbial communities (Nair and Ngouajio, 2012; Marinari et al., 2015; Detheridge et al., 2016). In this study, we found that cover crop treatments significantly increased the abundance of bacteria and fungi in 2020 (Figures 3A,B). This was consistent with the general observation that plant diversity has positive effects on soil microbial biomass due to the increases in soil carbon resources (Eisenhauer et al., 2010). Moreover, we also found that the abundance of bacteria and fungi in cover crop treatments in 2020 was higher than that in 2019 (Figures 3A,B). This might be partly explained by the time-lag response of soil biota to plant diversity changes (Eisenhauer et al., 2010, 2011). Soil microbiota have a significant effect on plant productivity in natural ecosystems (Wagg et al., 2014). Thus, we deduced that the difference of yield in different years might be related to the change of soil microbiota. Soil bacterial communities are sensitive to soil characteristics, especially to the soil pH (Kuramae et al., 2011; Shen et al., 2013). Similar result was found in our present study where the abundance of bacteria was mainly changed by soil pH (Figure 4A). Thus, our results suggest that the increased abundance of bacteria in the cover crop treatments compared with CK might be related to the higher pH.

High-throughput amplicon sequencing analysis in our study found that the Shannon index of the bacterial community only in the WB treatment was significantly higher than that of the CK in 2020 (Figure 5B), indicating our first hypothesis was not fully validated. Theoretically, higher plant diversity can be an efficient way to increase soil microbiota diversity due to the increase in soil habitat heterogeneity and carbon resource diversity (Eisenhauer et al., 2010, 2011), but some studies found that plant diversity have positive, neutral, or negative effects on soil microbe diversity (Wardle, 2006; Schlatter et al., 2015). It takes 4–6 years for soil biota to respond significantly to the changes in plant diversity (Eisenhauer et al., 2011), thus these inconsistent results may derive from the different duration of cover crops. Previous study found that multi-mixtures of grass and legumes improve microbial diversity when compared with single and control treatments (Vukicevich et al., 2016). Similarly, our results found that the bacterial diversity was higher in WB than in W. The possible reason could be that a mixture of high C/N cover crop (wheat) with legumes facilitates nutrient mineralization and provide a C source for diverse groups of soil organisms (Carrera et al., 2007; Dahal et al., 2020). MRT analysis indicated that bacterial diversity was mainly shaped by soil available P (Figure 4B). Long-term application of high phosphorus fertilizers to soil decreased the diversity of total bacteria (Liu et al., 2020b). In our research, the concentration of available P in CK treatment was higher than that in high P fertilizer treatment reported by Liu et al. (2020b). Thus, we also deduced that the higher Shannon index in the WB treatment was partly attributed to the lower soil available P.

### Effect of Cover Crops on the Composition of Bacterial Communities

In our study, the main phyla in the cover crop system were *Proteobacteria*, *Acidobacteria*, *Bacteroidetes*, *Actinobacteria*, and *Firmicutes* (Figure 6A), which are related to soil C degradation (DeAngelis et al., 2013; Trivedi et al., 2015). This result is consistent with the finding of Zheng et al. (2018a). Moreover, we found that *Firmicutes* were more abundant in the W and WRB treatments as compared to CK, indicating that they were the main decomposing bacteria of cover crop residues in the W and WRB treatments. This was also supported by an analysis conducted at the genus levels (the relative abundance of *Bacillus* spp. was increased in the W and WRB treatments relative to CK (Figure 7A).

The positive interactions between plant and microbe can result from the increasing of abundance of plant-beneficial microorganisms (Bever et al., 2012). Our high-throughput amplicon sequencing analysis showed that cover crop treatments increased the relative abundances of several bacterial taxa, which have plant-growth-promoting and/or pathogen-antagonistic potentials in pepper rhizosphere (Manzanera et al., 2015; Zheng et al., 2018b; Mun et al., 2020). For example, the relative abundances of *Streptomyces*, *Arthrobacter*, and *Bacillus* spp. were significantly increased in both the W and WRB treatments as compared to CK (Figure 7A). The relative abundance of *Pseudomonas* spp. which was lower than 0.5% in all treatments, was significantly increased in the WRB treatment as compared to CK (Supplementary Figure S1). Similarly, quantitative PCR found that W and WRB treatments significantly increased the abundance of *Bacillus* and *Pseudomonas* spp. in 2020 (Figures 3C,D). Wheat can release malic acid and benzoxazinoids in root exudates, which attract *Bacillus* and *Pseudomonas* spp., respectively (Fang et al., 2008; Berendsen et al., 2012). Therefore, the increase in the relative abundance of potential plant-beneficial bacteria in the W and WRB treatments might be partly attributed to the release of secondary metabolites from wheat root exudates or litter decomposition (Berendsen et al., 2012). The sensitivity of bacterial genera to changes in the soil environment varies, thus, their responses to soil environmental variables might differ (Sun et al., 2020). In our study, the higher pH and NO_3_^−^ content and lower C/N ratio in the W and WRB treatments might be another reason for the higher relative abundances of *Streptomyces*, *Arthrobacter*, and *Bacillus* spp., since the relative abundances of these three genera were positively related to the pH and soil NO_3_^−^ content, but negatively related to the C/N ratio (Figure 7B). In addition, we found that the relative abundance of *Lechevalieria* was higher in the R and WR treatments than in CK (Figure 7A). The antibiotic produced by the genus *Lechevalieria* has been found highly effective against plant fungal pathogens (Lee et al., 2004). Thus, we deduced that the R and WR treatments might enhance the resistance of plants to pathogens. This hypothesis is consistent with a previous study showing that the use of wild rocket as green manure decreased the severity of cucumber *Fusarium* wilt disease (Jin et al., 2019). The positive correlation between the relative abundance of *Lechevalieria* and soil pH (Figure 7B) indicated that increasing soil pH could be beneficial for its growth (Aslam et al., 2020).

The change of soil microbial community structure has been shown to be affected by substrate availability and quality associated with plant diversity (Nautiyal et al., 2010; Mbuthia et al., 2015). Therefore, cover crop mixtures as increasing species diversity would result in a significant shift in microbial community structure by producing a variety of substrates and increasing habitat heterogeneity (Spedding et al., 2004; Carrera et al., 2007). A previous study showed that the use of oats and mixtures containing oats as cover crops increased the accumulation of total N and SOC compared with brassica-only and legume-only treatments (Ghimire et al., 2019). In our study, the use of wheat as a cover crop by itself or mixed with faba bean might have provided a continuous food supply for microbial communities and promoted the growth of soil microbe (Thapa et al., 2021). This may explain the shifts in bacterial community structure under wheat treatment and mixture treatments containing wheat (e.g., W, WB, and WRB), relative to CK (Figure 8). However, we found that the community structure of WR differed to that of the W (Figure 8A), which possibly owing to the difference in the soil environment. Wild rocket as a member of the Brassicaceae family contain glucosinolates in their tissue, which are the precursor for the production of bioactive compounds isothiocyanates (White and Weil, 2010; Koide and Peoples, 2012). Isothiocyanates could alter bacterial community structure (Cohen et al., 2005). However, our results found that the community structure of R and mixtures containing R as cover crops were similar to that of CK (Figure 8). Similarly, Klose et al. (2006) did not observe apparent changes of soil microbial community structure after treating with methyl isothiocyanate. We suggest, this may be due to the short half-life time of these compounds and can disappear within a few days after their release into the soil environment (Motisi et al., 2009; Hanschen et al., 2015). Several studies have shown that soil physicochemical properties, especially soil pH, are important factors affecting soil bacterial community structures (Shen et al., 2013; Liu et al., 2014). Our present study found that W, WB, and WRB treatments had higher pH and lower NH_4_^+^ concentration compared with CK, which stems from continued N fertilization (especially NH_4_^+^-N) of the continuous soil and/or the release of H^+^ into the soil by nitrification (Yu et al., 2019). RDA and Mantel tests were performed to further confirm that soil pH and NH_4_^+^ were significantly correlated with the soil bacterial community composition, which supported our second hypothesis. These results were in accordance with the results of Yu et al. (2019). Thus, cover crop treatments likely altered soil bacterial community structure through their effects on the chemical properties of the soil. This conclusion is consistent with the results of previous studies showing that plants type might indirectly affect the bacterial community by altering the pH and N status of soil (Nielsen et al., 2010; Shen et al., 2013).

Indicator species are considered as ecological indicators of environmental changes, community type, and habitat conditions because of their niche preferences (Cáceres and Legendre, 2009). PCoA analysis showed that the bacterial community structure of W, WB, and WRB treatments were different from CK (Figure 8). In this study, there were 264 OTUs that differed among the eight treatments (Figure 10). Only eight OTUs were enriched solely in the CK. However, a total of 43, 51, and 26 OTUs were enriched solely in the W, WB, and WRB treatments, respectively. The indicator species in W (OTU2333, OTU2959, and OTU755) and WRB (OTU5821, OTU3068, and OTU3228) treatments were most closely related to *Pseudomonas*, *Bacillus*, and *Streptomyces* spp., respectively. Root exudates from wheat play key role in recruiting several species of the *Pseudomonas* and *Bacillus* taxa (Berendsen et al., 2012). For WB treatment, the indicator species OTU4588 was most closely related to *Gaiella* (Figure 10), which has been shown to play a key role in the resistance of strawberry plants against *F. oxysporum* (Zhao et al., 2019). These results suggest that cover crop treatments altered the bacterial community structure with shifts in the dominant genus, which have plant-growth-promoting and/or pathogen-antagonistic potentials in W, WB, and WRB treatments. The root exudates from wheat increases in beneficial microorganisms may be responsible for the enhanced promoting effect of changes in soil biota induced by cover crops on pepper seedling growth; however, additional work is needed to test this hypothesis.

## Conclusion

In this study, the effects of cover crops alone and their different combinations were evaluated on pepper yield, soil physicochemical properties, and soil bacterial communities in a continuous pepper cropping system. We found that the pepper yield was increased in the WR and WB in 2019 and 2020 and in the WRB in 2020. Cover crops increased the soil pH, decreased the concentrations of NH_4_^+^ and available phosphorus, and altered the bacterial abundance and community composition. A shift in certain bacterial taxa with mostly potential plant-beneficial bacteria specific to different cover crop combinations was observed. Moreover, soil pH was a strong indicator of these changes. Therefore, we conclude that mixing different cover crops could affect the soil environment differentially and regulate soil microbial community composition to benefit crop production. We also assumed that the enrichment of different taxa specific to different crop combination could be the result of differences in crop root exudates composition, which play critical role in rhizosphere assembly of microbial community. However, the assumption needs further study to underpin the detailed mechanism of how diversified cover crops regulate soil microbial community and increase crop yield.

## Data Availability Statement

The datasets presented in this study can be found in online repositories. The names of the repository/repositories and accession number can be found below: https://www.ncbi.nlm.nih.gov/, SRP326928.

## Author Contributions

FW contributed to design this experiment. HG and GT performed the experiment. HG analyzed the data and wrote the manuscript. MR revised the manuscript. All authors contributed to the article and approved the submitted version.

## Funding

This work was funded by National Key Research and Development Program of China (2019YFD1001900 and 2018YFD1000800), and Ministry of Finance, Ministry of Agriculture, and Rural Affairs, National Modern Industrial Technology System (CARS).

## Conflict of Interest

The authors declare that the research was conducted in the absence of any commercial or financial relationships that could be construed as a potential conflict of interest.

## Publisher’s Note

All claims expressed in this article are solely those of the authors and do not necessarily represent those of their affiliated organizations, or those of the publisher, the editors and the reviewers. Any product that may be evaluated in this article, or claim that may be made by its manufacturer, is not guaranteed or endorsed by the publisher.

## Supplementary Material

Supplementary Figure S1 The relative abundance of *Pseudomonas* spp. in different treatments. Different lowercase letters in each column denote significant differences among the samples (*p* < 0.05, HSD Tukey test). CK, no cover crop; W, wheat; R, wild rocket; B, faba bean; WR, wheat + wild rocket; WB, wheat + faba bean; RB, wild rocket + faba bean; and WRB, wheat + wild rocket + faba bean.
