# Supplementary material for: Cover Crop Species Composition Alters the Soil Bacterial Community in a Continuous Pepper Cropping System
Source: Front Microbiol. 2022 Jan 3;12:789034. doi: 10.3389/fmicb.2021.789034 (PMC8762165; doi:10.3389/fmicb.2021.789034)
Supplement: Supplementary file 1 [file Table_1.doc]

Table S1 Cover crop biomass under various cover crop treatments

| Treatment | May 2019 | October 2020 |
| --- | --- | --- |
| wheat (W) | 4.30±0.41 a | 3.30±0.13 a |
| faba bean (B) | 3.91±0.76 a | 3.48±0.23 a |
| wild rocket (R) | 4.00±0.38 a | 3.33±0.19 a |
| wheat+faba bean (WB) | 4.04±0.61 a | 3.37±0.46 a |
| wheat+wild rocket (WR) | 4.21±0.47 a | 3.41±0.61 a |
| wild rocket+faba bean (RB) | 4.20±0.06 a | 3.30±0.06 a |
| Wheat+wild rocket+faba bean (WRB) | 4.00±0.80 a | 3.33±0.29 a |
