# Supplementary material for: Cover Crop Species Composition Alters the Soil Bacterial Community in a Continuous Pepper Cropping System
Source: Front Microbiol. 2022 Jan 3;12:789034. doi: 10.3389/fmicb.2021.789034 (PMC8762165; doi:10.3389/fmicb.2021.789034)
Supplement: Supplementary file 2 [file Table_2.doc]

**Table S2** Correlation coefficients for relationships between pepper yield and soil physicochemical properties regardless of year

|  | pH | EC | NO_3_^-^ | NH_4_^+^ | TN | AP | AK | SOM | C/N |
| --- | --- | --- | --- | --- | --- | --- | --- | --- | --- |
| Pepper yield | 0.394** | -0.468** | -0.017 | -0.427** | 0.089 | -0.070 | 0.660** | 0.047 | -0.038 |

TN: soil total nitrogen; AP: available phosphorus; AK: available potassium; SOM: soil organic matter. (**p* < 0.05; ***p* < 0.01).
